# Comparison of Two Puncture Approaches in Robot‐Assisted Percutaneous Kyphoplasty for Upper Thoracic Osteoporotic Vertebral Compression Fractures

**DOI:** 10.1155/rrp/5131684

**Published:** 2026-10-25

**Authors:** Yongtao Yang, Wei Chen, Rujie Qin, Chunpeng Ren

**Affiliations:** ^1^ The Affiliated Lianyungang Hospital of Xuzhou Medical University Lianyungang Jiangsu China xzmc.edu.cn; ^2^ Lianyungang Municipal Oriental Hospital Lianyungang Jiangsu China

**Keywords:** costotransverse joint approach, orthopedic robot, osteoporotic vertebral compression fracture, percutaneous kyphoplasty (PKP), upper thoracic vertebra

## Abstract

**Objective:**

This study aimed to compare the clinical efficacy and safety of the robot‐assisted trans‐costotransverse joint approach and transpedicular approach in the treatment of upper thoracic single‐segment osteoporotic vertebral compression fractures (OVCFs).

**Methods:**

The data of 60 patients who underwent robot‐assisted percutaneous kyphoplasty (PKP) from October 2023 to January 2025 were retrospectively analyzed. According to the different surgical approaches, they were divided into two groups: trans‐costotransverse joint group (Group A *n* = 30) and transpedicular group (Group B *n* = 30). Perioperative indicators and clinical outcomes were compared.

**Results:**

The rate of satisfactory bone cement distribution was significantly higher in Group A than in Group B (90.0% vs. 66.7%, *p* = 0.028; OR = 4.50, 95% CI: 1.09–18.50), and the bone cement injection volume was greater (2.35 ± 0.34 mL vs. 2.13 ± 0.28 mL, *p* = 0.008), with mean difference (MD) = 0.22 mL (95% CI: 0.06–0.38 mL). The 1‐year follow‐up showed that the vertebral recollapse rate in Group A was significantly lower than that in Group B (3.3% vs. 20%, *p* = 0.044; OR = 0.14, 95% CI: 0.02–1.23). The incidence of bone cement leakage was lower in Group A than in Group B (6.7% vs. 26.7%, *p* = 0.038; OR = 0.20, 95% CI: 0.04–1.02). Although the VAS score, ODI indices, and local Cobb angle of the two groups were significantly improved compared with those before operation, there was no significant difference between the two groups.

**Conclusion:**

In this retrospective analysis of patients with upper thoracic OVCF, robot‐assisted trans‐costotransverse joint PKP may result in a more favorable distribution of bone cement and improved short‐ to medium‐term vertebral stability as compared with the transpedicular approach. This procedure shows acceptable safety profile, though further prospective studies are required to validate its clinical benefit.

## 1. Introduction

Osteoporotic vertebral compression fracture (OVCF) is a significant health concern in the elderly population. As the population ages, its prevalence continues to rise [1, 2]. Statistics indicate that the annual increase of OVCF is about 4%‐5%, and the incidence rate is more than 850 cases per 100,000 people in 5 years after age adjustment [3]. Patients often present with persistent back pain, kyphosis, limited mobility, and other symptoms, and the quality of life is seriously affected [4]. Percutaneous vertebroplasty (PVP) and percutaneous kyphoplasty (PKP) are the main minimally invasive procedures for the treatment of OVCF. They can quickly relieve pain and reconstruct vertebral morphology, with minimal tissue trauma and reduced blood loss. They have been widely used in clinical practice [5, 6].

Performing PKP in the upper thoracic spine (T1–T6) presents unique anatomical challenges, including narrow pedicles, short transverse processes, and costovertebral articulations. Conventional freehand puncture techniques struggle to achieve accurate needle trajectory control in this region, thereby increasing the risk of complications such as trajectory deviation, bone cement extravasation, and neural injury [7, 8]. In recent years, the application of orthopedic surgical robot has provided new ideas for solving the above problems. The robot‐assisted system can complete three‐dimensional path planning before surgery, realize real‐time navigation during surgery, complete high‐precision puncture trajectory design, and effectively improve puncture accuracy and surgical safety [9]. Existing studies have shown that compared with traditional fluoroscopy‐assisted PKP, robot‐assisted PKP can significantly reduce the risk of bone cement leakage, reduce the radiation exposure of both doctors and patients during surgery, and achieve better effects in correcting kyphosis Cobb angle and restoring vertebral height [10, 11].

The clinical puncture approach of PKP is currently mainly through the pedicle approach. When it is applied to the upper thoracic segment, due to the small width of the pedicle and the large inclination angle, unilateral puncture often fails to achieve bilateral balanced distribution of bone cement in the vertebral body [12]. The costotransverse joint approach can bypass the narrow pedicle structure and directly enter the lateral area of the vertebral body through the costotransverse joint. This path allows a more lateral puncture angle, which facilitates wider cement diffusion, optimizes distribution, and reduces the risk of leakage [13]. Despite advances in robot‐assisted spinal surgery, few studies have directly compared these two puncture trajectories for upper thoracic OVCF. Specifically, sufficient clinical evidence remains lacking regarding whether either approach offers superior protection against postoperative vertebral recollapse while maintaining comparable functional outcomes. Through retrospective analysis, this study compared the clinical effects of the robot‐assisted unilateral trans‐costotransverse joint approach and the transpedicular approach for PKP in the treatment of upper thoracic OVCFs and provided a reference for clinical selection of safe and effective minimally invasive surgical approaches.

## 2. Materials and Methods

This study retrospectively analyzed 60 patients with OVCF of the upper thoracic spine (T1–T6) from October 2023 to January 2025 who met the inclusion criteria. According to the actual puncture approach implemented during surgery, they were divided into 30 patients in the robot‐assisted unilateral PKP via the costotransverse joint (Group A) and 30 patients in the robot‐assisted unilateral PKP via the pedicle (Group B). All patients were followed for 12–18 months after surgery. The study protocol has been approved by the Institutional Medical Ethics Committee (Approval No.: JS‐20250227001‐01). All patients provided written informed consent before surgery.

Inclusion criteria were as follows: (1) age 60 to 85 years, of any gender; (2) bone mineral density T‐score ≤ −2.5; (3) single‐segment compression fracture of a vertebral body located in the upper thoracic spine (T1–T6); (4) MRI showing bone marrow edema within the affected vertebral body (high T_2_ signal); and (5) disease duration ≤ 6 weeks with severe impact on daily life.

Exclusion criteria were as follows: (1) vertebral burst fractures; (2) pathological fractures; (3) severe comorbidities precluding tolerance of anesthesia and surgery; (4) definite spinal cord compression or spinal canal stenosis requiring decompressive surgery; and (5) inability to complete standardized imaging follow‐up or presence of incomplete follow‐up data.

### 2.1. Surgical Equipment

The TiRobot orthopedic surgical robot system (Beijing Tianzhihang Medical Co., Ltd., China) was employed in this study. This system consists of three core modules: a six‐degree‐of‐freedom robotic arm, an optical real‐time tracking subsystem, and a main control workstation for image reconstruction and surgical planning. The standardized intraoperative workflow contained three key phases: registration, trajectory planning, and real‐time navigation. (1) Registration: After placement of the optical locator in the surgical field, intraoperative three‐dimensional computed tomography (CT) scanning was performed. Image datasets were imported to the main control workstation for automatic image to patient registration, establishing spatial correspondence between virtual reconstructed images and the actual patient anatomy. (2) Trajectory planning: On the reconstructed 3D thoracic spinal model, the surgeon delineated the puncture entry point, trajectory angle, and target intravertebral endpoint for either the trans‐costotransverse joint approach or transpedicular approach, as described above. Trajectory simulation was executed to avoid collision with vital adjacent structures. (3) Intraoperative navigation: Under continuous optical tracking, the robotic arm automatically moved to the preplanned spatial position and provided a stable guiding cannula. The surgeon manually inserted the guide needle along the robotic aligned trajectory. Real‐time optical monitoring was maintained throughout puncture; if substantial patient movement occurred, reregistration would be triggered to correct navigation deviation. Other operative instruments included puncture needles, working cannulas, and balloon catheters (Shanghai Kinetic Medical Co., Ltd., China). Bone cement was purchased from Tecres S.P.A. (Italy).

### 2.2. Surgical Method

The schematic diagram of the two puncture trajectories is presented in Figure 1.

**FIGURE 1:**
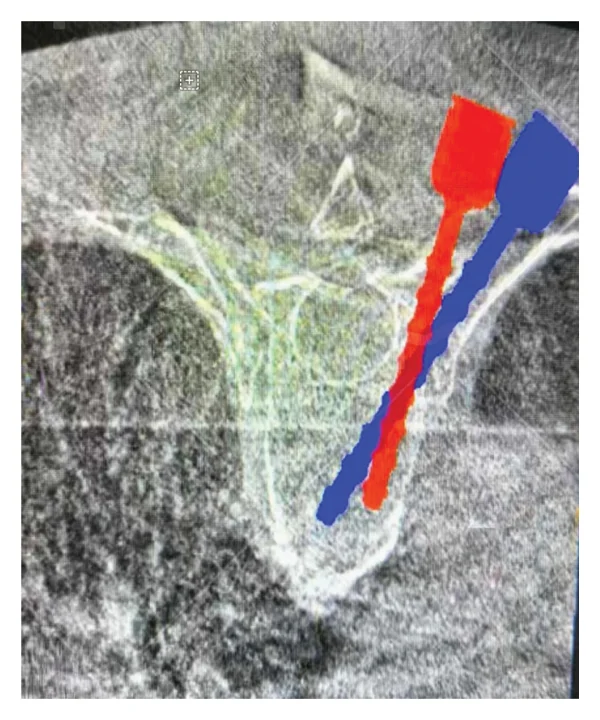
Schematic diagram of two puncture trajectories (the blue line represents the trans‐costotransverse joint approach, and the red line represents the transpedicular approach).

Group A: Patients underwent general anesthesia to mitigate involuntary movement caused by pain or anxiety, which could compromise intraoperative 3D image acquisition and navigation accuracy. Subsequently, patients were placed in the prone position. The surgical site was sterilized and draped. After the procedure, the intelligent navigation robot was connected to the C‐arm machine. The operator positioned the optical locator in the surgical field, selected one intact vertebral body above and one below the target puncture segment as reference points, and performed three‐dimensional CT scanning. Once the images were transmitted to the robot system, the operator planned the trajectory targeting the costotransverse joint approach. The anatomical landmark for this approach was the junction of the transverse process, rib neck, and superior inferior costotransverse articular capsule. The puncture entry point was located at the posterolateral surface of the costotransverse joint. The planned abduction angle ranged from 35° to 50° relative to the mid‐sagittal plane, allowing the needle to cross the vertebral midline and reach the anterior‐middle one‐third of the vertebral body, without traversing the pedicle lumen. The system automatically calculated the required needle insertion depth. After the system confirmed the plan, the operator activated the robotic arm, which automatically positioned the puncture tool along the planned trajectory. Under navigation guidance, the operator inserted the guide needle and then sequentially placed the working cannula and the bone drill. Anteroposterior fluoroscopy showed the needle tip had crossed the midline of the vertebral body, and lateral fluoroscopy showed the puncture needle lay within the pedicle shadow. A balloon was then positioned at the vertebral body center and slowly inflated until vertebral height was restored or the pressure reached the preset threshold. After confirming satisfactory reduction, the balloon was removed. The bone cement was prepared to the stringy stage and then slowly injected using the bone cement syringe. Fluoroscopy with the C‐arm machine was performed after every 0.3 mL of injection to ensure that the cement did not breach the posterior edge of the vertebral body or leak into the intervertebral space or venous system. Injection was stopped when the cement reached the posterior edge of the vertebral body or when leakage occurred. After the cement had fully hardened, the instruments were removed. The incision was closed with a single stitch, compressed locally, and bandaged.

Group B: Preoperative preparation and anesthesia were the same. Robot trajectory planning was performed for the standard transpedicular approach. The anatomical landmark was the conventional posterolateral pedicle entry point (the junction of the transverse process and the lateral border of the superior articular process). The planned medial inclination angle was 10°–20° relative to the mid‐sagittal plane, consistent with the natural anatomical abduction angle of upper‐thoracic pedicles. The needle passed through the pedicle canal and terminated at the anterior one‐third of the vertebral body. The insertion depth was automatically generated by the robotic planning system. After the system confirmed there were no errors, the operator activated the robotic arm, which automatically positioned the puncture tool along the planned path. Under navigation guidance, the operator inserted the guide needle, then the working cannula and bone drill, and stopped at the anterior one‐third of the vertebral body. Subsequently, balloon dilation and bone cement injection were performed, following the same procedure and operation standards as in Group A. After the operation, disinfection, suturing, and dressing were completed.

Postoperative management: Patients were encouraged to mobilize with brace support 2 h after surgery. Three‐dimensional CT of the thoracic spine was reexamined 24 h after surgery to assess bone cement filling and detect any leakage, and the local Cobb angle was measured on reexamined anteroposterior and lateral thoracic spine radiographs. All patients began systematic antiosteoporosis treatment after surgery.

### 2.3. Observation Outcomes

Primary outcomes included the following: the volume of bone cement injected, the distribution of bone cement, bone cement leakage, and recollapse of the injured vertebra. (1) Bone cement injection volume: It was recorded intraoperatively during PKP. (2) Bone cement distribution: It was evaluated by anteroposterior and lateral radiographs of the thoracic spine performed at 24 h after surgery. The vertebral body was divided into left and right halves along the central line of the vertebral body. If the bone cement crossed the midline of the vertebral body on the anteroposterior and lateral images, it was judged to be well distributed; if the bone cement did not cross the midline in any projection direction, it was judged to be generally distributed [14]. (3) Bone cement leakage: It was evaluated by thoracic three‐dimensional CT performed at 24 h after surgery. Cement leakage was categorized according to leakage location (paravertebral, intervertebral, intraspinal, or intravascular). (4) Recollapse of the injured vertebra: It was defined as an increase of local Cobb angle > 3° at the 12‐month follow‐up compared with the postoperative 24 h thoracic anteroposterior and lateral radiographs [15].

Secondary outcomes included the following: Visual Analog Scale (VAS) score [16], Oswestry Disability Index (ODI) [17], and improvement of the local Cobb angle. These measures were recorded before surgery and at 3 days, 1 month, 3 months, and 12 months after surgery.

Quality control for radiological measurements: Considering the anatomical overlap of clavicle, scapula, and ribs that may impair plain film image quality in the upper thoracic region, all radiological evaluations were implemented with the following quality control measures. (1) Bone cement leakage and cement distribution were judged on postoperative 24‐h 3D‐CT rather than two‐dimensional fluoroscopy, to eliminate the superimposition artifacts of upper thoracic osseous structures. (2) All radiographic Cobb angle measurements were independently completed by two experienced spinal surgeons who were blinded to group allocation. Each radiograph was measured twice at an interval of 2 weeks. The mean value of the two observers’ repeated measurements was adopted for statistical analysis. The intraobserver reliability and interobserver reliability were calculated using the two‐way random‐effects absolute‐agreement intraclass correlation coefficient (ICC). For local Cobb angle measurements on upper thoracic radiographs, the intraobserver ICC was 0.84 (95% CI: 0.76–0.90), and the interobserver ICC was 0.77 (95% CI: 0.67–0.85). For categorical assessments of bone cement leakage and cement distribution grade based on postoperative 3D‐CT, the intraobserver ICC was 0.91 (95% CI: 0.86–0.95), and the interobserver ICC was 0.88 (95% CI: 0.81–0.93). ICC values > 0.75 were considered as satisfactory measurement reliability. Images with poor visualization were excluded, and repeated radiography was arranged for the patient.

### 2.4. Statistical Methods

SPSS 26.0 statistical software (IBM Corp, Chicago, IL, USA) was used to analyze the research data. Firstly, the Shapiro–Wilk normality test was performed on all continuous variables. Data following a normal distribution were expressed as mean ± standard deviation (SD) and analyzed by an independent sample *t*‐test. The measurement data between the two groups were compared by independent sample *t* test. Categorical data were presented as counts (percentages) and analyzed using the chi‐square test or Fisher’s exact test. Repeated measures analysis of variance was used to compare the preoperative and postoperative time periods within each group. *p* < 0.05 was considered statistically significant.

## 3. Results

All procedures were completed successfully in both groups. Baseline characteristics—including age, sex, fracture level, follow‐up duration, bone mineral density T‐score, body mass index (BMI), operative time, intraoperative blood loss, fluoroscopy dose, and comorbidities—were comparable between the two groups (all *p* > 0.05; Table 1).

**TABLE 1:** Baseline data of patients in the two groups.

	Group A (*n* = 30)	Group B (*n* = 30)	*t*(*X* ^2^)	*p*
Age (years)	69.80 ± 6.50	70.70 ± 5.21	−0.591	0.557
Sex (male/female, cases)	9/21	8/22	0.082	0.774
Fracture site (T1/T2/T3/T4/T5/T6)	2/2/4/6/8/8	1/2/5/5/8/9	0.594	0.988
Follow‐up time (months)	15.22 ± 1.44	15.10 ± 1.36	0.322	0.748
Bone mineral density T‐score	−3.09 ± 0.35	−3.08 ± 0.26	0.211	0.833
BMI (Kg/m^2^)	22.66 ± 3.35	22.88 ± 2.54	−0.286	0.776
Operation time (min)	51.93 ± 5.51	52.13 ± 3.81	−0.163	0.871
Intraoperative blood loss (ml)	25.10 ± 3.95	25.33 ± 5.27	−0.194	0.847
Fluoroscopy dose (Gy·cm^2^)	198.70 ± 14.25	200.27 ± 12.30	−0.456	0.650
Comorbid conditions (cases)				
Hypertension	14 (47%)	15 (50%)	0.067	0.796
Diabetes	11 (37%)	8 (27%)	0.693	0.405
Coronary heart disease	7 (23%)	9 (30%)	0.341	0.559

*Note:* Data are expressed as mean ± SD or absolute number (percentage).

Abbreviation: BMI, body mass index.

Satisfactory bone cement distribution was achieved in 27 patients (90.0%) in Group A compared with 20 patients (66.7%) in Group B. There was a statistically significant difference in the rate of satisfactory bone cement distribution between the two groups (*p* = 0.028). The rate of satisfactory bone cement distribution of Group A was better, and the odds ratio (OR) was 4.50 (95% CI: 1.09–18.50). In terms of bone cement injection volume, Group A was (2.35 ± 0.34) mL, and Group B was (2.13 ± 0.28) mL. The injection volume of Group A was significantly higher than that in Group B (*p* = 0.008). The mean difference (MD) between the two groups was 0.22 mL (95% CI: 0.06–0.38 mL).

Vertebral recollapse occurred in 1 patient (3.3%) in Group A and 6 cases (20%) in Group B at 1 year postoperatively. The rate of recollapse in Group A was lower than that in Group B (*p* = 0.044). The OR was 0.14 (95% CI: 0.02–1.23). In terms of intraoperative bone cement leakage, there were 2 cases (6.7%) in Group A and 8 cases (26.7%) in Group B. The rate of bone cement leakage in Group A was lower than that in Group B (*p* = 0.038). The OR was 0.20 (95% CI: 0.04–1.02). All leakages were confined to the paravertebral or intervertebral space area, without obvious clinical symptoms and special treatment. There were no serious complications such as pneumothorax or hemothorax in both groups. One patient had increased intercostal neuralgia postoperatively in Group A, and the symptoms were relieved after 1 month (Table 2).

**TABLE 2:** Comparison of main indicators between the two groups.

	Group A (*n* = 30)	Group B (*n* = 30)	OR/MD (95% CI)	*p*
Bone cement distribution quality (good/general, cases)	27/3	20/10	4.50/(1.09–18.50)	0.028
Bone cement injection volume (ml)	2.35 ± 0.34	2.13 ± 0.28	0.22/(0.06–0.38)	0.008
Vertebral recollapse (cases)	1 (3.3%)	6 (20%)	0.14/(0.02–1.23)	0.044
Bone cement leakage (cases)	2 (6.7%)	8 (26.7%)	0.20/(0.04–1.02)	0.038

*Note:* Data are expressed as mean ± SD or absolute number (percentage).

The VAS score, the ODI indices, and the local Cobb angle of the two groups were significantly improved postoperatively, and the difference was statistically significant (*p* < 0.001), but there were no significant differences in the interaction effect or between‐group effect (*p* > 0.05) (Table 3 and Figure 2).

**TABLE 3:** Comparison of VAS, ODI indices, and local Cobb angle between two groups at different time points.

	Group A (*n* = 30)	Group B (*n* = 30)
VAS		
Preoperative	6.57 ± 0.94	6.37 ± 0.99
3 d	2.63 ± 0.61	2.43 ± 0.50
1 m	1.90 ± 0.48	1.87 ± 0.51
3 m	1.67 ± 0.66	1.47 ± 0.57
12 m	1.33 ± 0.48	1.27 ± 0.45
*F*_time_/*F*_time∗group_/*F*_group_	676.868/0.524/1.734	
*P*_time_/*P*_time∗group_/*P*_group_	< 0.001/0.719/0.193	
ODI indices (%)		
Preoperative	77.54 ± 6.19	80.21 ± 7.21
3 d	36.72 ± 3.43	36.87 ± 3.12
1 m	23.92 ± 3.95	24.81 ± 3.75
3 m	23.39 ± 4.26	23.99 ± 4.12
12 m	22.42 ± 4.31	22.99 ± 4.12
*F*_time_/*F*_time∗group_/*F*_group_	1256.080/0.784/1.899	
*P*_time_/*P*_time∗group_/*P*_group_	< 0.001/0.540/0.175	
Local Cobb angle (°)		
Preoperative	14.18 ± 3.83	13.87 ± 3.74
3 d	7.95 ± 0.76	8.16 ± 0.82
1 m	8.21 ± 0.75	8.38 ± 0.74
3 m	8.38 ± 0.69	8.46 ± 0.71
12 m	9.26 ± 0.77	9.57 ± 0.86
*F*_time_/*F*_time∗group_/*F*_group_	67.774/1.780/0.144	
*P*_time_/*P*_time∗group_/*P*_group_	< 0.001/0.146/0.705	

*Note:* Repeated‐measures ANOVA showed significant intragroup differences among different time points for Visual Analog Scale (VAS) score, Oswestry Disability Index (ODI), and local Cobb angle in both groups (all *p* < 0.001); there were no significant differences in the interaction effect or between‐group effect (*p* > 0.05). Data are expressed as mean ± SD. 3 d: 3 days postoperatively; 1 m: 1‐month postoperatively; 3 m: 3‐month postoperatively; 12 m: 12‐month postoperatively.

**FIGURE 2:**
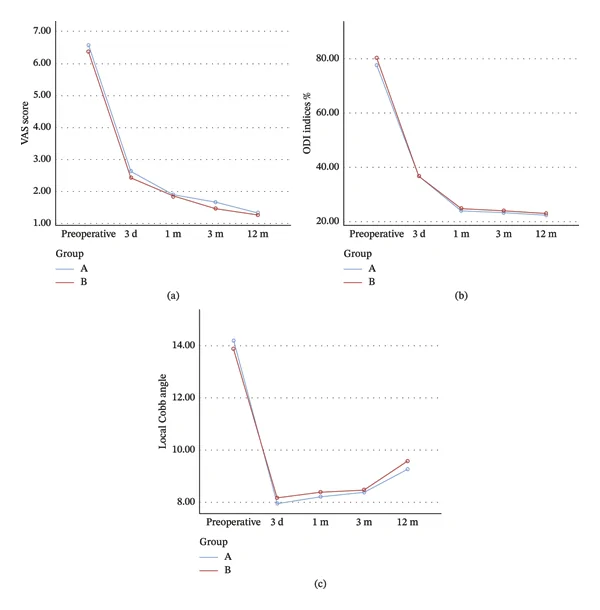
Line charts of VAS score, ODI indices, and local Cobb angle at different time points for two groups. (a) shows VAS score; (b) shows ODI indices (%); (c) shows local Cobb angle (°). Data are expressed as mean ± standard deviation. 3d: 3 days postoperatively; 1 m: 1‐month postoperatively; 3 m: 3‐month postoperatively; 12 m: 12‐month postoperatively.

### 3.1. Typical Case

A 63‐year‐old female patient presented to the hospital more than one hour after sustaining trauma with pain and discomfort in the chest and back. Thoracic spine MRI showed bone marrow edema of the T5 vertebral body. Robot‐assisted PKP via the trans‐costotransverse joint approach was performed. The pre‐ and posttreatment conditions are shown in Figure 3.

**FIGURE 3:**
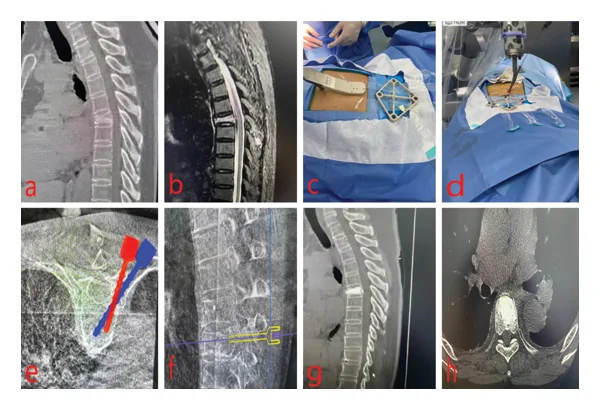
(a, b) show three‐dimensional CT and MRI of the thoracic spine before surgery, demonstrating a fracture of the T5 vertebral body; (c, d) show intraoperative positioning and puncture; (e, f) show intraoperative approach planning; (g, h) show three‐dimensional CT of the thoracic spine postoperatively, demonstrating satisfactory distribution of bone cement.

## 4. Discussion

This study retrospectively compared the efficacy of the robot‐assisted unilateral trans‐costotransverse joint approach and transpedicular approach PKP in the treatment of upper thoracic OVCF. In this retrospective comparison of two robot‐assisted PKP puncture trajectories for upper thoracic OVCF, the trans‐costotransverse joint approach demonstrated advantages in cement distribution, cement injection volume, cement leakage prevention, and midterm anti‐recollapse performance. However, no intergroup differences were found in pain relief, functional improvement, or local kyphosis correction. The robot‐assisted trans‐costotransverse joint approach therefore represents a favorable option for upper thoracic OVCF given its improved cement‐related safety and midterm biomechanical performance.

Our findings reaffirmed the value of robotic assistance in spinal surgery, particularly in technically demanding regions such as the upper thoracic spine. Compared with traditional free‐hand puncture, the robotic system avoided injury to critical structures by using preoperative three‐dimensional image planning and intraoperative precise registration, thereby reducing the risk of puncture‐related complications. No serious complications occurred in either group, and one case of transient intercostal neuralgia in Group A further aligns with multiple studies of robot‐assisted PVP and PKP, which reported higher puncture accuracy and lower complication rates [18, 19]. However, to contextualize our contributions relative to prior work (e.g., Yuan et al. [9] and Liu et al. [18]), it is important to note a key distinction. Those studies primarily established the superiority of robot‐assisted over fluoroscopy‐assisted PKP. In contrast, our study presupposes the efficacy of robotic assistance and addresses a more granular question: which puncture trajectory yields superior outcomes under robotic guidance? By utilizing the robot to eliminate operator experience and free‐hand errors, our study design isolates the effect of the trajectory itself. Therefore, the incremental contribution of this study is not merely to confirm the feasibility of robotic assistance in the upper thoracic spine; rather, it provides direct evidence that the trans‐costotransverse joint approach is a superior technique compared to the transpedicular approach when both are robot‐assisted. Our findings advance the field by optimizing the specific surgical strategy within robot‐assisted PKP, demonstrating that trajectory choice significantly impacts cement distribution and long‐term biomechanical stability.

Bone cement leakage remains the most prevalent complication of PKP. While minor anterolateral leakage is typically asymptomatic, intraspinal extravasation may cause sensorimotor deficits necessitating urgent surgical decompression. Intravascular leakage carries a risk of pulmonary embolism and can have severe consequences [20, 21]. This study found that the leakage rate was lower with the trans‐costotransverse joint approach. Possible explanations are as follows. The abduction angle of the upper thoracic pedicle is only 10°–20°, so the puncture needle often cannot reach past the vertebral body midline. In contrast, the puncture angle of the trans‐costotransverse joint approach can reach up to 50° [22]. Navigation guidance allowed the needle to be advanced more controllably to the anterior‐middle one‐third of the vertebral body, reducing the risk of breaching the lateral or anterior vertebral wall. Better cement distribution also reduced leakage from weak points created by uneven intravertebral cement pressure. This study showed that the rate of satisfactory bone cement distribution through the trans‐costotransverse joint approach was 90.0%, which was significantly higher than that of the transpedicular approach (66.7%). This result was consistent with the anatomical and clinical findings from previous studies on the robot‐assisted lateral approach. A simulation study by Wang et al. [23] demonstrated that the transverse process‐pedicle approach had a larger puncture abduction angle than the traditional transpedicular approach, and this advantage continued from the upper lumbar segment to the middle thoracic segment. According to the comparative study of robot‐assisted unilateral and bilateral PKP, Tao et al. [24] proposed that robot‐assisted techniques can optimize the distribution pattern of bone cement, and the unilateral approach can achieve a similar distribution effect as the bilateral approach, which further supports the conclusion of this study. In terms of bone cement injection volume, the mean injection volume in the trans‐costotransverse joint group was 2.35 mL, slightly higher than 2.13 mL in the transpedicular group. The injection volume of the two groups was lower than the average 4.7 mL reported by Qian et al. [25]. The difference was mainly attributed to the significantly smaller volume of the upper thoracic vertebrae compared to that of the lumbar vertebrae, aligning with the anatomical characteristics of different spinal segments. There was a significant difference in the distribution of bone cement between the two groups, but there was no significant difference in the improvement of the VAS score, the ODI indices, and the local Cobb angle, which was consistent with the findings reported in the literature [12, 24]. The analgesic mechanism of PKP mainly includes stabilizing microfractures in the vertebral body after bone cement solidification, destroying pain sensing nerve endings, and improving the local biomechanical state [26]. Both approaches can effectively restore the mechanical stability of the spine.

This study found that there were significant differences in the rate of recollapse of injured vertebrae across different surgical paths. The recollapse rate in Group A was lower than that in Group B (3.3% vs. 20.0%). The underlying reasons can be explained by the anatomical and biomechanical differences between the two puncture trajectories. The trans‐costotransverse joint approach allows a larger abduction angle of up to 50°, enabling the needle tip to cross the midline. This trajectory facilitates more uniform cement dispersion throughout the vertebral body. A larger volume of bone cement and a more uniform distribution can form a stronger internal support structure, improve biomechanical stability, and disperse load stress [27]. A more uniform distribution may increase the contact area between bone cement and surrounding trabecular bone, thereby enhancing stability [28]. During follow‐up, vertebral bodies exhibiting an uneven distribution of bone cement are prone to stress concentration, which may eventually lead to endplate collapse and a secondary loss of vertebral height [29]. Postoperative collapse of the injured vertebra can not only cause the loss of early correction angle and vertebral height but also lead to intractable chest and back pain, aggravation of kyphosis, and even neurological impairment in severe cases, which directly affects the long‐term quality of life of patients [19]. Furthermore, decreasing the incidence of recollapse can lower the need for revision surgeries such as secondary PKP and posterior internal fixation, which not only mitigates secondary trauma and psychological burden for patients but also reduces overall medical costs. The advantage of the trans‐costotransverse joint approach in reducing the rate of recollapse of injured vertebrae, in addition to statistical differences, also has clear clinical and health‐economic value.

While this study offers direct comparative evidence for two robot‐assisted approaches, it has some limitations. First, the sample size was modest (*n* = 30 per group). As a retrospective study utilizing archived clinical data, no a priori power analysis was conducted. While post hoc power calculation suggested that our sample could detect moderate to large effect sizes for primary outcomes, small‐magnitude intergroup differences might remain undetected due to limited statistical power. The maximum postoperative follow‐up of 18 months was insufficient to evaluate the long‐term efficacy and postoperative complications. Second, this study only included single‐segment upper thoracic spine OVCF; it remains to be verified whether it applies to multisegment upper thoracic spine fractures and lower thoracic spine fractures. Retrospective design may lead to selection bias, and prospective randomized trials will be needed in the future to confirm our findings. Third, the high purchase and maintenance costs of the robot‐assisted system may limit its widespread adoption in primary hospitals. Fourth, while this study utilized postoperative three‐dimensional CT to evaluate the distribution and leakage of bone cement, more advanced methods, such as finite element analysis, were not employed to quantify biomechanical parameters. Fifth, all procedures were performed under general anesthesia. Although general anesthesia can improve the accuracy and safety of surgery, it poses additional perioperative risks to elderly patients.

## 5. Conclusion

This retrospective study demonstrates that robot‐assisted PKP via the trans‐costotransverse joint approach resulted in superior bone cement distribution and enhanced midterm vertebral stability, with comparable functional recovery compared with the transpedicular approach. Given the limitations of a retrospective single‐center design, these observations should be interpreted cautiously, and further prospective studies are needed to verify our conclusions for clinical decision‐making.

## Author Contributions

Yongtao Yang and Wei Chen analyzed and interpreted the data. Chunpeng Ren and Rujie Qin performed the surgery. Yongtao Yang wrote the main manuscript text.

## Funding

This study was supported by the Medical Technology Support Project of Lianyungang First People’s Hospital (Grant No. YK202502).

## Disclosure

All authors read and approved the final manuscript.

## Ethics Statement

This study was approved by the Ethics Committee of the Affiliated Lianyungang Hospital of Xuzhou Medical University (Approval No. JS‐20250227001–01). All patients provided written informed consent before surgery.

## Conflicts of Interest

The authors declare no conflicts of interest.

## Data Availability

The data that support the findings of this study are available from the corresponding authors upon reasonable request.
